# IFI16 Induced by p53 Activates the NF‐κB Pathway to Counteract Cisplatin‐Induced Apoptosis in Cervical Cancer Cells

**DOI:** 10.1111/jcmm.70728

**Published:** 2025-08-06

**Authors:** Lili Zhong, Jiaxin Li, Jianfeng Zhong, Yifan Zhang, Hang Qi, Huimei Yu, Xin Li

**Affiliations:** ^1^ Jilin Provincial Key Laboratory on Molecular and Chemical Genetics The Second Hospital of Jilin University Changchun Jilin China; ^2^ Department of Pathophysiology, College of Basic Medical Sciences Jilin University Changchun Jilin China; ^3^ Department of Basic Medical Sciences Changchun Medical College Changchun Jilin China; ^4^ Clinical Laboratory The Second Hospital of Jilin University Changchun Jilin China; ^5^ Department of Breast Surgery The Second Hospital of Jilin University Changchun Jilin China

**Keywords:** cervical cancer, cisplatin, IFI16, NF‐κB, p53, STING

## Abstract

Cervical cancer ranks as the second most prevalent cancer among women worldwide, and the primary treatment for advanced cases involves cisplatin‐based chemotherapy. However, the duration of cisplatin treatment is typically short, with a median survival rate of approximately 1 year. This highlights the urgent need to enhance our understanding of cisplatin's mechanism of action in cervical cancer treatment. Our findings demonstrate that p53 induces the nuclear translocation of IFI16, leading to activation of the NF‐κB signalling pathway. This activation plays a crucial role in protecting cervical cancer cells against cisplatin‐induced apoptosis. The activation of NF‐κB is independent of STING, which is a downstream molecule of IFI16. STING signalling activation by cisplatin may not be associated with cisplatin‐induced apoptosis. To further validate this tumour‐promoting effect of IFI16 during cisplatin therapy, we established a subcutaneous implantation tumour model using mouse cervical cancer (U14) cells and conducted additional in vitro experiments. We examined the role and mechanism of IFI16 in cisplatin treatment of cervical cancer. The role of IFI16 in cervical cancer progression deserves further study. Targeted inhibition of IFI16 may be a new way to increase cisplatin sensitivity of cervical cancer cells.

## 1 Introduction

Cervical cancer is the second most common cancer in women worldwide, with an estimated 600,000 cases and more than 300,000 deaths per year [1]. Cisplatin is still the main treatment for advanced cervical cancer. Although the tumour suppressor gene p53, activated by cisplatin‐induced DNA damage, has been a concern for a long time [2], the targeted regulatory pathways of p53 are extensive and complicated. Recently, researchers have paid more attention to the molecular pathway and function of p53, which are involved in cell survival and apoptosis escape in addition to tumour suppressor genes [3]. While p53 exerts tumour‐suppressive functions, mTOR, Ras, NF‐κB and other signalling pathways are also affected by p53 to promote tumour development [4], thereby influencing p53‐induced tumour cell death. Therefore, exploring the activation mechanism of p53‐related pro‐survival signals can provide clues to elucidate the mechanism for enhancing cisplatin sensitivity of cervical cancer cells.

Exposure to chemotherapy agents or radiation has been observed to enhance NF‐κB activity in different cell lines, stimulating the transcription of proliferation‐regulating genes (such as cyclin D1 and C‐MYC) and promoting multiple pro‐tumour functions [5, 6, 7]. Some data suggest that the use of bortezomib, sulfasalazine, lapatinib or other NF‐κB inhibitors can act as chemotherapy sensitizers by inhibiting the NF‐κB pathway in the treatment of progressive metastatic breast cancer [8, 9, 10]. In addition, in cervical cancer HeLa cells, interferon β and low‐dose cisplatin have synergistic inhibitory effects on human cervical cancer cells [11]. Since interferon beta inhibits NF‐κB/p‐Akt signalling, this suggests that inhibition of the NF‐κB/p‐Akt signalling pathway may play a key role in the anti‐cancer effects of combination therapy. In cervical cancer, the strategy of cisplatin in combination with NF‐κB inhibitors is promising. Hundreds of compounds have been reported to inhibit NF‐κB, but their clinical efficacy has so far been unsatisfactory except in certain types of lymphoma and leukaemia [12], possibly due to the lack of tumour cell specificity in most current NF‐κB targeting strategies. It is suggested that targeting upstream regulatory molecules of NF‐κB has therapeutic potential in cervical cancer.

Previous studies have suggested that dsDNA damage can cause the activation of the NF‐κB pathway [13, 14], and p53 plays an important role in the activation of NF‐κB induced by dsDNA damage [15, 16]. A cooperative relationship between p53 and NF‐κB (rather than the expected antagonism) has been reported in some cells. For example, after human embryonic kidney cells (HEK) are stimulated by etoposide or mouse leukaemia cells (AML‐4) are stimulated by plasmids, p53 induces pro‐apoptotic protein (PIDD) with death domain to activate NF‐κB to regulate apoptosis [13, 17]. After alveolar type II epithelial cells (AEC II) were stimulated by BCG infection, p53 cooperates with MDM2 to negatively regulate CBP, thereby up‐regulating the NF‐κB signalling pathway. p53 and NF‐κB pathways have been shown to synergistically participate in the immune regulation of AECII cells against *Mycobacterium tuberculosis* (MTB) infection [18]. It is suggested that the binding of p53 to different proteins can affect the function of NF‐κB through different pathways. Therefore, determining the role of p53 on NF‐κB may improve the understanding of the mechanism of cisplatin in the treatment of cervical cancer.

Interferon‐induced factor IFI16 has been described as a tumour suppressor protein, which is associated with DNA damage response factors such as p53 and plays a role in promoting cell senescence [19]. Many previous studies have shown that IFI16 is also a viral DNA sensor, and IFI16/STING/NF‐κB signalling is involved in regulating innate immune response and apoptosis [20, 21, 22]. Therefore, taking IFI16 as the entry point to explore the mechanism of IFI16's involvement in p53 regulation of NF‐κB has clinical significance to further clarify the mechanism of cisplatin treatment of cervical cancer cells.

## 2 Materials and Methods

### 2.1 Reagents and Antibodies

Cisplatin and 3‐(4, 5‐Dimethylthiazol‐2‐yl)‐2, 5‐diphenyltetrazolium bromide (MTT) were purchased from Sigma‐Aldrich (St. Louis, MO, USA). H‐151 was purchased from MCE (USA). The FITC Annexin V Apoptosis Detection Kit was purchased from BD Biosciences (State of New Jersey, USA). ViaFect Transfection Reagent was purchased from Promega (Madison, MI, USA). Nuclear and cytoplasmic protein extraction kit was purchased from Beyotime (China). The following antibodies were used: anti‐β‐actin, anti‐LaminA/C, anti‐α‐tubulin, anti‐p53, anti‐p65, anti‐IFI16, anti‐STING (Proteintech, Chicago, IL, USA); anti‐Phospho‐STING (Ser366), anti‐p65 (Santa Cruz Biotechnology, Santa Cruz, CA, USA); anti‐Cleaved‐Caspase3, anti‐Phospho‐Histone H2A.X (Cell Signalling Technology, Beverly, Massachusetts, USA).

### 2.2 Cell Lines and Cell Culture

Human cervical cancer (HeLa) cells and mouse cervical cancer (U14) cells were purchased from iCell Bioscience Inc. (Shanghai, China). HeLa cells were cultured in 1640 medium (Gibco Life Technologies, Carlsbad, CA, USA) containing 10% fetal bovine serum (Invitrogen, Carlsbad, CA, USA). U14 cells were cultured in high‐glucose DMEM medium (Gibco Life Technologies, Carlsbad, CA, USA) containing 10% fetal bovine serum (Gibco Life Technologies, Carlsbad, CA, USA). Both cells were cultured in a cell incubator at 5% CO_2_ and 37°C.

### 2.3 Transfection

1. The cells were placed in a 6‐well plate, and the experiment was carried out when the cell density reached 70%–90% under the microscope.
2. 2–5 μg plasmid/siRNA + 250 μL serum‐free 1640 medium was incubated for 5 min, then 6–10 μL transfection reagent + 250 μL serum‐free medium was incubated together for 20 min. After mixing well, 1640 culture solution containing 10% FBS was added into a 6‐well plate and cells were cultured in a cell incubator for 24 h.
3. Pmp53 plasmid (Jilin University, Jilin, China) was constructed by the Department of Pathophysiology of Jilin University to restore the function of p53 [23]. The plasmid overexpressed wild‐type p53 and inhibited MDM2 expression through siMDM2. MDM2 is a negative feedback regulator of p53, so Pmp53 is more active than p53. Therefore, the Pmp53 plasmid was used to overexpress p53 in HeLa cells in this study.
4. siRNA‐IFI16 was ordered from GenePharma (Shanghai, China). The sequences are as follows:

siCtrl

Forward: UUCUCCGAACGUGUCACGUTT

Reverse: ACGUGACACGUUCGGAGAATT

siIFI16‐1 (human)

Forward: CCAAGCAGCAGUUUCUUAATT

Reverse: UUAAGAAACUGCUGCUUGGTT

siIFI16‐2 (human)

Forward: GCUUUGCUCACAAACUAAATT

Reverse: UUUAGUUUGUGAGCAAAGCTT

siIFI16‐1 (mouse)

Forward: GAAUGUUUCAUGCUACCGUTT

Reverse: ACGGUAGCAUGAAACAUUCTT

siIFI16‐2 (mouse)

Forward: CCAACAAUGGUUAUCUCAATT

Reverse: UUGAGAUAACCAUUGUUGGTT

### 2.4 Cell Viability Assays

8 × 10^3^ cells were seeded in 96‐well plates in each well. After 36 h, cells were treated with different concentrations of cisplatin after transfection. The MTT assay was used to evaluate cell viability. We measured the absorbance at 570 nm using a Vmax Microplate Reader (Molecular Devices LLC, Sunnyvale, CA, USA).

### 2.5 Western Blot Analysis

Proteins were quantified using BCA reagent (Biotime Biotechnology, Shanghai, China) before western blot. Proteins were then separated in 10% sodium dodecyl sulfate polyacrylamide gel (Willget Biotech, Shanghai, China) and transferred onto nitrocellulose membrane (Millipore, Shanghai, China). Then the membrane was blocked by 5% defatted milk for 1 h and added with primary antibody at 4°C overnight. Horse radish peroxidase (HRP)‐conjugated secondary antibodies were incubated (Proteintech, Chicago, IL, USA) according to the manufacturer's instructions. Then, immunodetection was performed using ECL reagent (Thermo Fisher Scientific) and visualised by a Syngene Bio Imaging (Synoptics, Cambridge, UK).

### 2.6 Immunofluorescence Staining and Confocal Laser Microscopy

Cells were washed and fixed in 4% (w/v) paraformaldehyde/PBS for 20 min and subjected to proteinase K digestion for 1 min. Then, they were permeabilised with 0.1% Triton X‐100 for 15 min. After blocking with bovine serum albumen for 30 min, cells were incubated with primary antibody overnight at 4°C. The next day, they were stained with FITC/Texas Red‐conjugated secondary antibodies (1:200 dilution; Proteintech, Chicago, IL, USA) for 30 min in the dark. Finally, cells were treated with Hoechst 33342/H_2_O (1 lg/mL) for 2 min, and images were acquired by an Olympus FV1000 confocal laser microscope.

### 2.7 Flow Cytometry

Cells (25 × 10^4^ cells/well) were seeded in 6‐well plates. After exposure to different treatments, cells were collected and performed Annexin‐V FITC/PI staining according to the manufacturer's instructions. Samples were analysed by Accuri C6 Flow Cytometry (BD Biosciences, Franklin Lakes, NJ, USA).

### 2.8 Quantitative Real‐Time PCR

Quantitative real‐time PCR was done by using the MX300P instrument (Agilent, USA) followed by a 2‐step PCR protocol. The primer sequences are as follows:

Primers for p21 (human)

Forward: CGATGGAACTTCGACTTTGTCA

Reverse: GCACAAGGGTACAAGACAGTG

Primers for IL‐6 (human)

Forward: TACAAAAGTCCTGATCCAGTTC

Reverse: AAGAAGGAATGCCCATTAAC

Primers for IFN‐β (human)

Forward: GCTTGGATTCCTACAAAGAAGCA

Reverse: ATAGATGGTCAATGCGGCGTC

Primers for Cyclin D1 (human)

Forward:GCTGCGAAGTGGAAACCATC

Reverse: CCTCCTTCTGCACACATTTGAA

Primers for Actin (human)

Forward: TGAAGAAGAACCGAGACTACCC

Reverse: TCCAGACGGTATTTGTCATCCT

The relative expression was calculated by Δ*Ct* among different experimental groups normalised to ACTB expression.

### 2.9 Mouse Subcutaneous Transplantation Tumour Model

The 6‐week‐old female C57BL/6J mice were purchased from Vital River (Peking, China) and had been fed for 1 week to adapt to the new environment. U14 cells (2 × 10^6^ each) were injected subcutaneously into the right axilla of the mice. When the tumour volume reached about 200 mm^3^, the mice were randomly divided into four groups. For the control group, 80 μL PBS was injected every 2 days. For the negative non‐target siRNA combined with cisplatin treatment group, the mixture of 15 μg negative non‐target siRNA (dissolved in 50 μL PBS) and 30 μL transfection reagent was injected at multiple points in the tumour every 2 days. For the siIFI16‐1/2 combined with cisplatin treatment group, intratumour injection of a mixture of 15 μg 2′‐o‐Me modified siIFI16 1/2 (dissolved in 50 μL PBS) and 30 μL transfection reagents was performed at multiple points every 2 days. The blank control group received intraperitoneal injection of 100 μL normal saline the next day. The negative non‐target siRNA combined with cisplatin treatment group and siIFI16‐1/2 combined with cisplatin treatment group received intraperitoneal injection of cisplatin the next day (cisplatin was 3 mg/kg, dissolved in 100 μL normal saline). The weight and tumour size of the mice were measured daily. The mice were euthanised after 1 week, and the subcutaneous tumours were weighed and photographed. All mice were raised in the pathogen‐free Animal Laboratory of Jilin University, and all animal studies were conducted in accordance with the protocol approved by the Animal Ethics Committee of Jilin University.

### 2.10 Statistical Analyses

Statistical analysis was performed using GraphPad Prism 9.1 (La Jolla, CA, USA). All the data are presented as means ± SD and carried out using the Student's *t*‐test. *p* < 0.05 was considered statistically significant.

## 3 Results

### 3.1 Cisplatin‐Induced DNA Damage Led to an Increase in p53 Protein Expression and Induced Apoptosis in Human Cervical Cancer Cells

**FIGURE 1:**
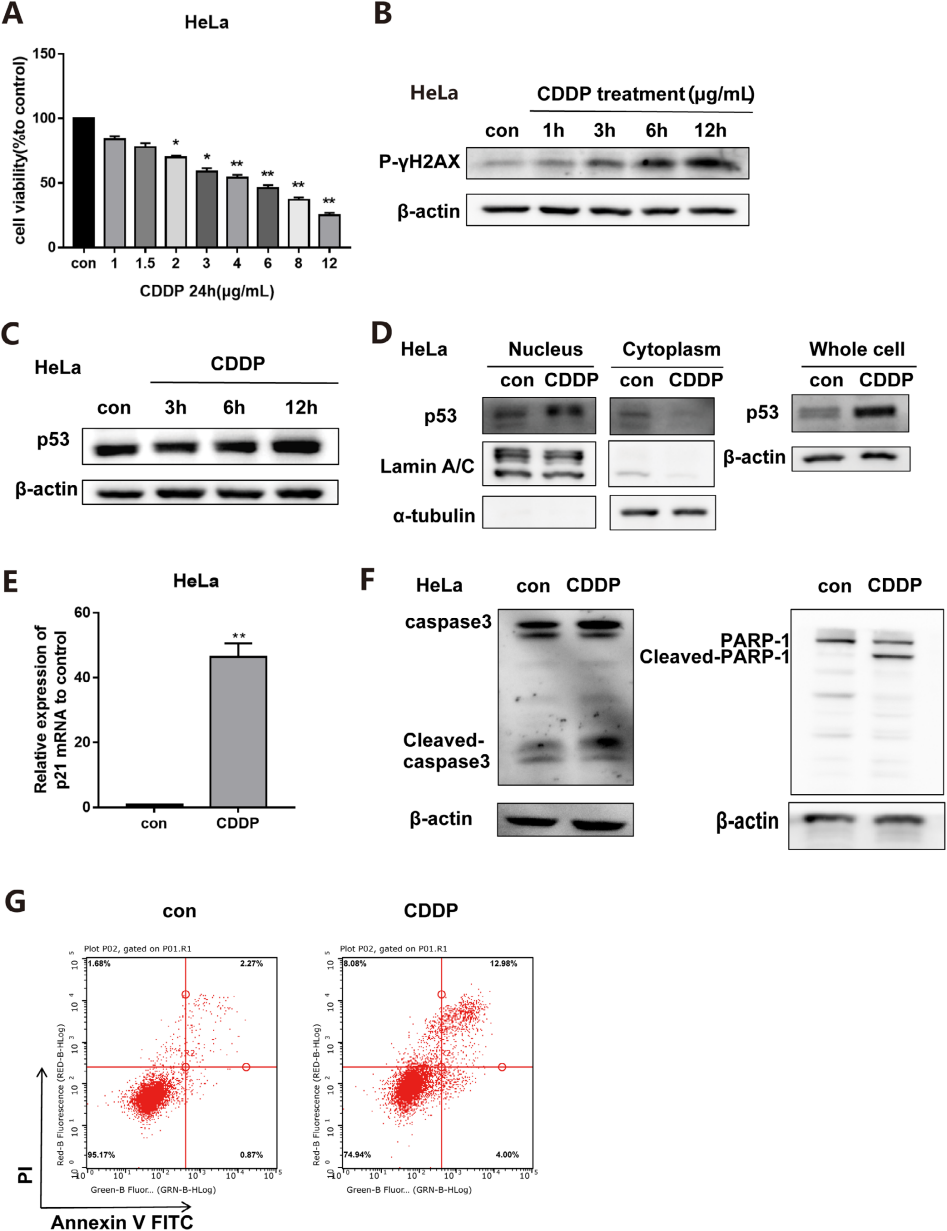
DNA damage induced by cisplatin increased p53 protein expression and induced apoptosis of human cervical cancer cells. (A) The cell activity of human cervical cancer cells HeLa was detected by MTT colourimetry. (B) The protein expression of γH2A.X in HeLa cells was detected by Western Blot. (C) The changes in p53 protein expression in HeLa cells were detected by Western Blot at different stages of cisplatin treatment (3 h, 6 h, 12 h). (D) After 12 h of cisplatin treatment, the expression of p53 in the nucleus, cytoplasm, and whole cell protein of HeLa cells was detected by Western Blot. (E) p21 mRNA expression in HeLa cells was detected by qPCR after 12 h of cisplatin treatment. (F) Western Blot analysis of Cleaved‐caspase3 and Cleaved‐PARP‐1 protein expression in HeLa cells. (G) The apoptosis level of HeLa cells was detected by flow cytometry. (CDDP: 4 μg/mL, data described as mean ± SD, *n* = 3, *p < 0.05, **p < 0.01)

To establish a model of DNA damage in HeLa cells, we treated them with cisplatin, a first‐line chemotherapy drug for cervical cancer, and assessed cell viability using the MTT assay (Figure 1A). It was observed that the protein expression of γ‐H2AX increased over time in the cisplatin treatment group, with a significant increase after 12 h of treatment (Figure 1B), indicating that cisplatin caused DNA damage in HeLa cells at a non‐lethal dose (4 μg/mL). Additionally, treating human cervical cancer HeLa cells with cisplatin for 12 h resulted in a significant increase in p53 protein expression (Figure 1C), nuclear translocation of p53 (Figure 1D), and upregulation of mRNA expression levels of the downstream gene p21 (Figure 1E). The expression levels of apoptotic protein cleaved‐caspase3 and cleaved PARP‐1 were also elevated upon cisplatin treatment (Figure 1F), leading to a significantly increased rate of apoptosis (Figure 1G). These findings suggest that cisplatin induces nuclear accumulation and activation of p53, enabling its transcriptional activity and promoting apoptosis in cervical cancer cells. This result is consistent with the current theory on the mechanism of cisplatin.

### 3.2 p53 Activates the NF‐κB Pathway and Promotes IFI16 Expression

**FIGURE 2:**
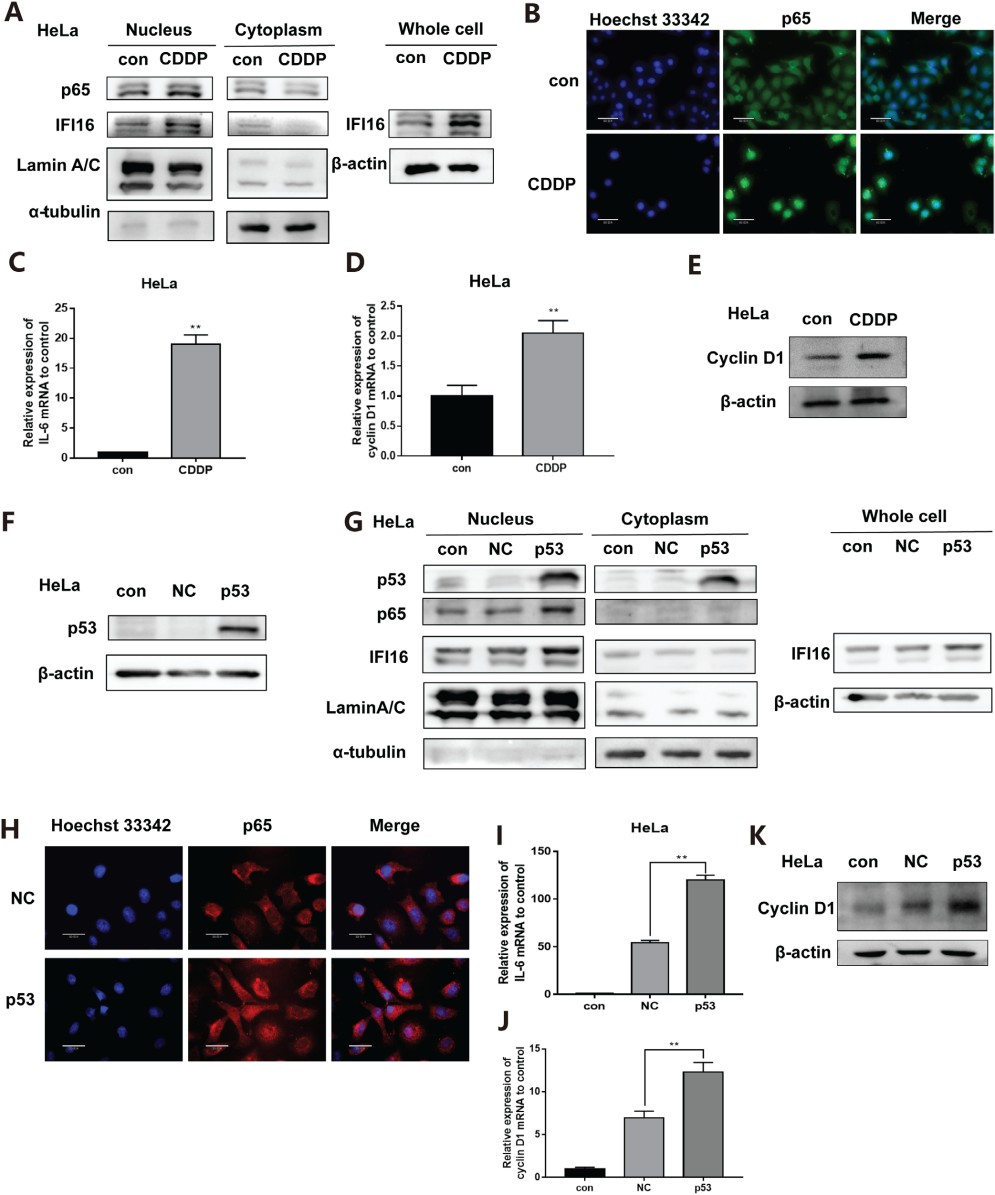
p53 activates the NF‐κB pathway and promotes IFI16 expression. (1) HeLa cells treated with cisplatin for 12 h: (A) The protein expression changes of p65 and IFI16 in the nucleus and cytoplasm of HeLa cells and the protein expression of IFI16 in the whole cells were detected by Western Blot. (B) The subcellular localisation of p65 in HeLa cells was detected by immunofluorescence (scale = 30 μm). (C) The expression level of IL‐6 mRNA in HeLa cells was detected by qPCR. (D) Changes of Cyclin D1 mRNA expression levels. (E) The changes in the expression level of Cyclin D1 protein. (2) Overexpression of p53: (F) Western Blot assay was used to detect the transfection efficiency of Pmp53 plasmid. (G) Western Blot assay was used to detect the changes of protein expression of p53, p65 and IFI16 in the nucleus and cytoplasm of HeLa cells, and the protein expression of IFI16 in HeLa cells in whole cells. (H) The subcellular localisation of p65 in HeLa cells was detected by immunofluorescence (scale = 60 μm). (I) The expression level of IL‐6 mRNA in HeLa cells was detected by qPCR. (J) The expression level of Cyclin D1 mRNA was detected by qPCR. (K) The changes in the expression level of Cyclin D1 protein. (CDDP: 4 μg/mL, data described as mean ± SD (*n* = 3), **p < 0.01).

In order to explore the effects of cisplatin on the expression and transcriptional activity of IFI16 and NF‐κB pathway‐related proteins, the nuclear and cytoplasmic proteins were isolated and extracted from HeLa cells after 12 h stimulation with cisplatin. The results showed that after cisplatin treatment, the level of p65 protein in the nucleus was significantly increased (Figure 2A,B), and the transcription levels of the target genes of the NF‐κB pathway IL‐6 (Figure 2C) and CyclinD1 (Figure 2D) were also significantly increased. The protein expression level of Cyclin D1 also increased (Figure 2E). This result is consistent with current knowledge about the activation of the typical NF‐κB pathway, which leads to phosphorylation of IκB, subsequent degradation and release of p65 into the nucleus, inducing transcription of genes such as IL‐6 and CyclinD1. Meanwhile, the expression level of IFI16 in both nuclear and whole‐cell proteins was increased after cisplatin treatment (Figure 2A). It is suggested that the NF‐κB pathway is activated and the expression of IFI16 is increased with obvious nucleation during the apoptosis of human cervical cancer cells induced by cisplatin.

In order to further verify the activation of the NF‐κB pathway specifically induced by p53, the Pmp53 plasmid was constructed to increase the expression of p53 protein (Figure 2F) and restore the function of p53. 24 h after the transfection of the Pmp53 plasmid into HeLa cells, nuclear protein and cytoplasmic protein were isolated. After the overexpression of p53, nuclear p65 protein was significantly increased (Figure 2G), mRNA expression levels of IL‐6 (Figure 2I) and Cyclin D1 (Figure 2J) were significantly increased, and the protein expression level of Cyclin D1 also increased (Figure 2K). Nuclear IFI16 protein expression was increased (Figure 2G). Combined with the results of the activation of the NF‐κB pathway during the apoptosis of human cervical cancer cells induced by cisplatin, p53 is the upstream molecule that causes the activation of the NF‐κB pathway and the nucleation of IFI16 in human cervical cancer HeLa cells under cisplatin stimulation.

### 3.3 Knockdown of IFI16 Inhibits NF‐κB Pathway and Increases the Sensitivity of Human Cervical Cancer Cells to Cisplatin

**FIGURE 3:**
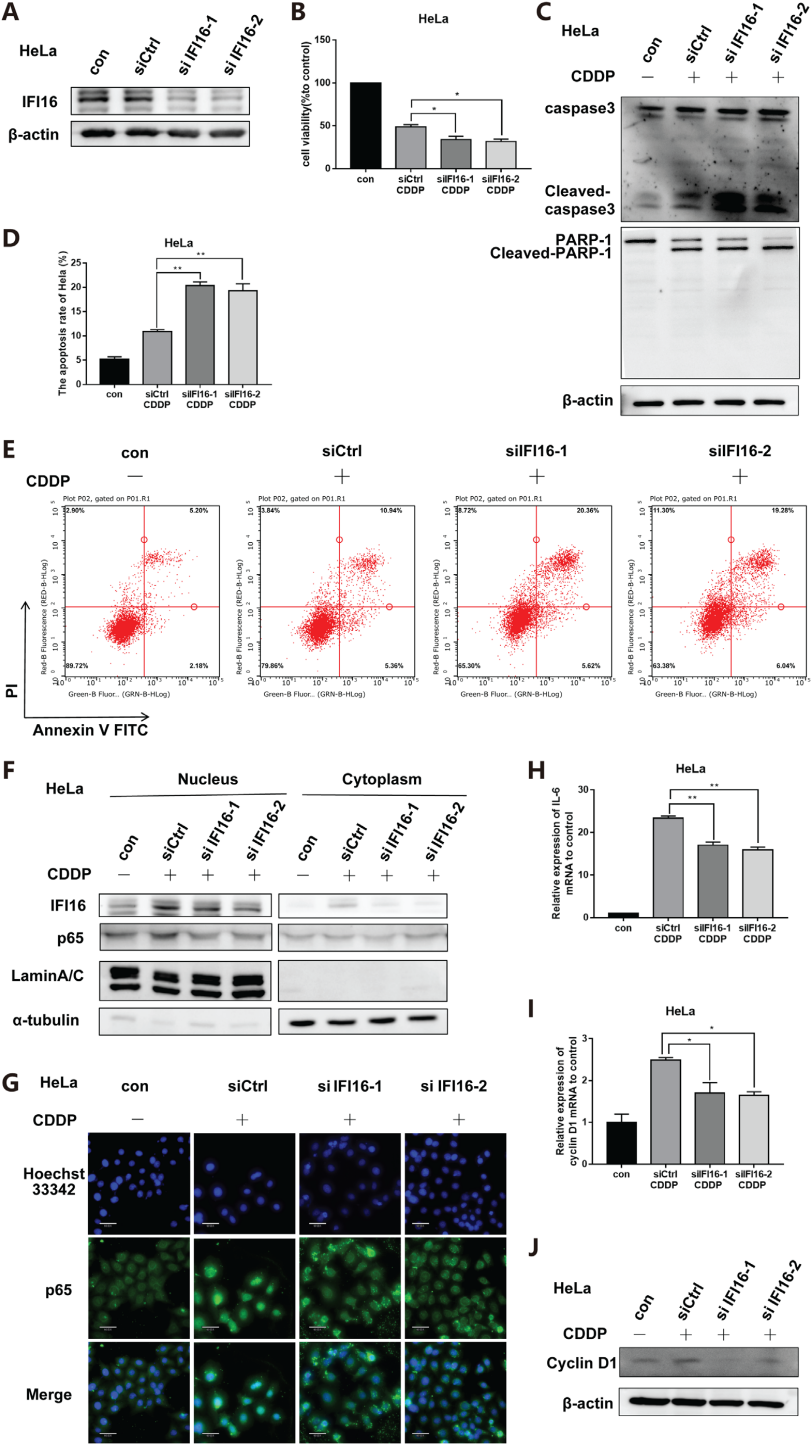
Knockdown of IFI16 inhibited NF‐κB pathway and increased the sensitivity of human cervical cancer cells to cisplatin. (A) siRNA‐IFI16 was transfected into HeLa cells, and the changes of IFI16 protein expression were detected by Western Blot. After transfection with siRNA‐IFI16, HeLa cells were treated with cisplatin for 12 h. (B) The cell activity of HeLa was detected by MTT colourimetry. (C) Western Blot assay was used to detect the expression of Cleaved‐caspase3 and Cleaved‐PARP‐1 protein in HeLa cells. (D) The apoptosis level of HeLa was detected by flow cytometry. (E) Quantitative analysis of the D‐chart. (F) Western Blot assay was used to detect the changes of p65 protein expression in HeLa cell nucleus. (G) The subcellular localisation of HeLa cell p65 was detected by immunofluorescence (scale = 30 μm). (H) The mRNA expression of IL‐6 in HeLa cells was detected by qPCR. (I) The expression of Cyclin D1 mRNA in HeLa cells was detected by qPCR. (J) Changes in the expression level of Cyclin D1 protein. (CDDP: 4 μg/mL, data described as mean ± SD (*n* = 3), *p < 0.05, **p < 0.01).

We constructed siRNA‐IFI16 to knock down IFI16 protein expression in human cervical cancer cell HeLa (Figure 3A). To explore the role of IFI16 in cisplatin‐induced apoptosis of cervical cancer cells, we stimulated HeLa cells with cisplatin (4 μg/mL) for 12 h after knockdown of IFI16. We observed that under cisplatin, knockdown of IFI16 resulted in a significant decrease in cell activity (Figure 3B) and an increase in apoptosis level (Figure 3C–E), indicating that IFI16 exerts anti‐apoptotic effects under cisplatin treatment.

Subsequently, to explore whether IFI16 can resist cisplatin‐induced apoptosis by activating the NF‐κB signalling pathway, we examined the degree of activation of the NF‐κB signalling pathway after IFI16 knockdown. The results showed that the expression level of p65 protein in the nucleus was significantly decreased (Figure 3F,G). Transcription levels of the NF‐κB pathway target genes IL‐6 and Cyclin D1 were also reduced (Figure 3H,I). The expression level of Cyclin D1 protein decreased (Figure 3J). The results indicated that IFI16 knockdown inhibited the activation of the NF‐κB signalling pathway induced by cisplatin, suggesting that IFI16 is the main molecule in the activation of the NF‐κB pathway against the apoptosis induced by cisplatin.

### 3.4 Knockdown of IFI16 Enhanced the Cisplatin Sensitivity of Subcutaneous Implantation Tumor of Cervical Cancer in Mice

In order to explore the in vivo effect of inhibiting IFI16 combined with cisplatin, a subcutaneous implantation tumour model of mouse cervical cancer cells was constructed. Mouse cervical cancer U14 cells were injected subcutaneously into the right underarm of female C57BL/6J mice. When the tumour volume reached about 200 mm^3^, 12 mice with tumours of the same size were randomly divided into four groups. They were the blank control group (con group), the negative non‐target siRNA combined with cisplatin treatment group (siCtrl + CDDP group), and siIFI16‐1/2 combined with cisplatin treatment group (siIFI1‐1/2 + CDDP group). For the control group, 80 μLPBS was injected every 2 days. For the negative non‐target siRNA combined with cisplatin treatment group, the mixture of 15 μg negative non‐target siRNA (dissolved in 50 μL PBS) and 30 μL RNAFit transfection reagent was injected at multiple points in the tumour every 2 days. For the siIFI16‐1/2 combined with cisplatin treatment group, intra‐tumour injection of a mixture of 15 μg 2′‐o‐Me modified siIFI16‐1/2 (dissolved in 50 μL PBS) and 30 μL RNAFit transfection reagents was performed at multiple points every 2 days. The blank control group received intraperitoneal injection of normal saline the next day, and the negative non‐target siRNA combined with cisplatin treatment group and siIFI16‐1/2 combined with cisplatin treatment group received intraperitoneal injection of cisplatin the next day (cisplatin was 3 mg/kg, dissolved in 100 μL normal saline). The status of the mice was observed every day, and the weight and tumour size of the mice were measured. The mice were euthanised after 1 week, and the subcutaneous tumours were weighed and photographed.

**FIGURE 4:**
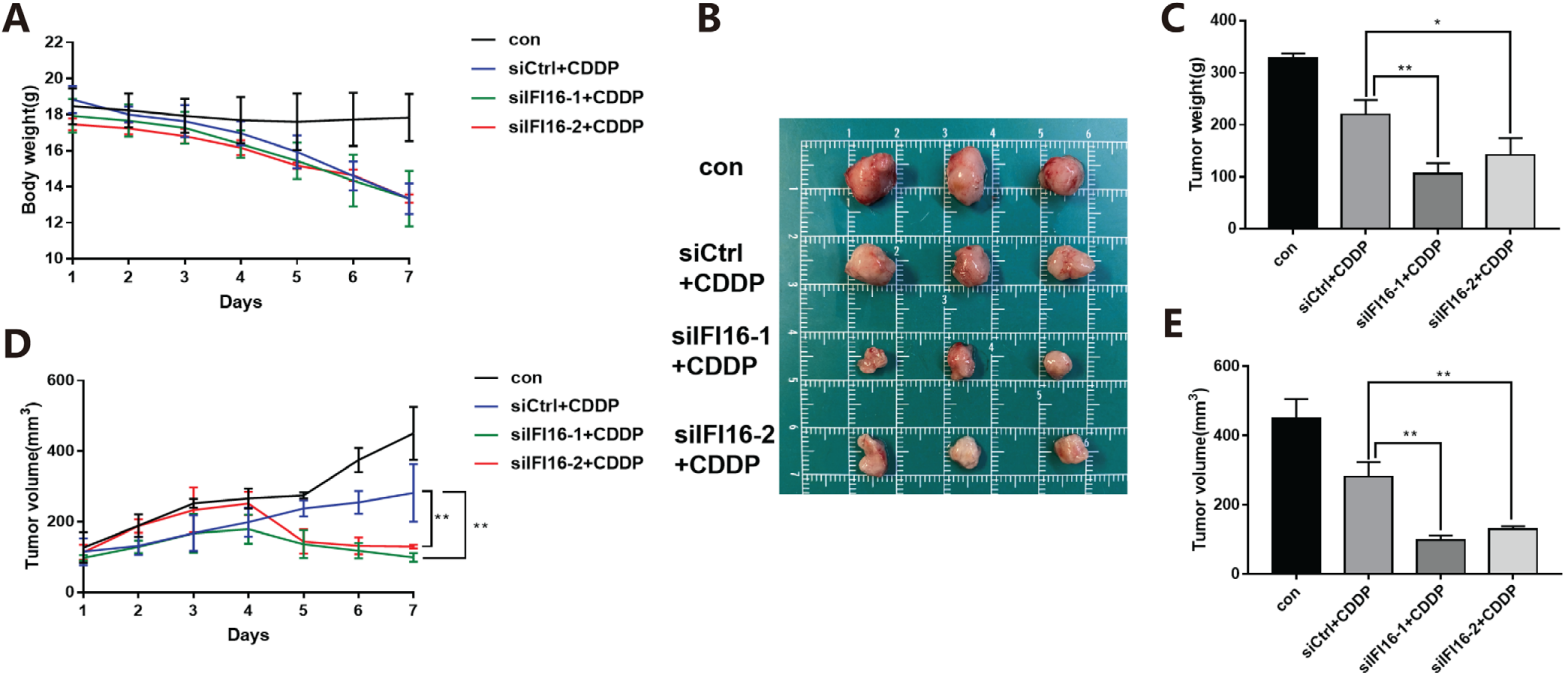
Effects of inhibition of IFI16 combined with cisplatin on mouse cervical cancer cell implantation tumours verified in vivo. (A) Inhibiting weight changes in C57BL/6J mice during IFI16 combined with cisplatin treatment. (B) Tumour tissue in vitro of C57BL/6J mice after IFI16 inhibition combined with cisplatin treatment. (C) Tumour termination weight comparison in C57BL/6J mice after IFI16 inhibition combined with cisplatin treatment. (D) Comparison of tumour termination volume in C57BL/6J mice treated with IFI16 inhibition combined with cisplatin. (E) Inhibition of tumour volume changes in C57BL/6J mice during IFI16 combined with cisplatin treatment. (CDDP: 3 mg/kg, data described as mean ± SD, *n* = 3, compared with siCtrl + CDDP, *p < 0.05, **p < 0.01).

The results showed that there was no significant change in body weight in the blank control group, and the body weight of mice in the negative non‐target siRNA combined with cisplatin treatment group and the siIFI16‐1/2 combined with cisplatin treatment group gradually decreased from Day 4, with no significant difference in the downward trend (Figure 4A). Compared with the negative non‐target siRNA combined with cisplatin treatment group, the volume, weight and growth rate of cervical cancer subcutaneous implant tumours in the siIFI16‐1/2 combined with cisplatin treatment group were further decreased (Figure 4B–E). The results suggested that inhibiting IFI16 enhanced the cisplatin sensitivity of subcutaneous implantation tumour of cervical cancer in mice.

### 3.5 IFI16/NF‐κB Resistance to Cisplatin Induced Apoptosis Was Not Associated With STING Signalling

Although it is controversial that cisplatin and other chemotherapy drugs can induce an immune response of tumour cells experiments have found that STING recruits and activates downstream TBK1 kinase after the activation of intracellular DNA signal, and STING Ser366 is phosphorylated by TBK1 to initiate cascade signal transduction [24, 25]. Therefore, the phosphorylated‐STING (p‐STING)/STING ratio represents the activation of the STING signalling pathway. As a DNA‐binding protein, IFI16 can cooperate with cGAS to sense cytoplasmic DNA in human keratinocytes and macrophages and activate the NF‐κB signalling pathway through the innate immune signal STING/TBK1 pathway [21].

**FIGURE 5:**
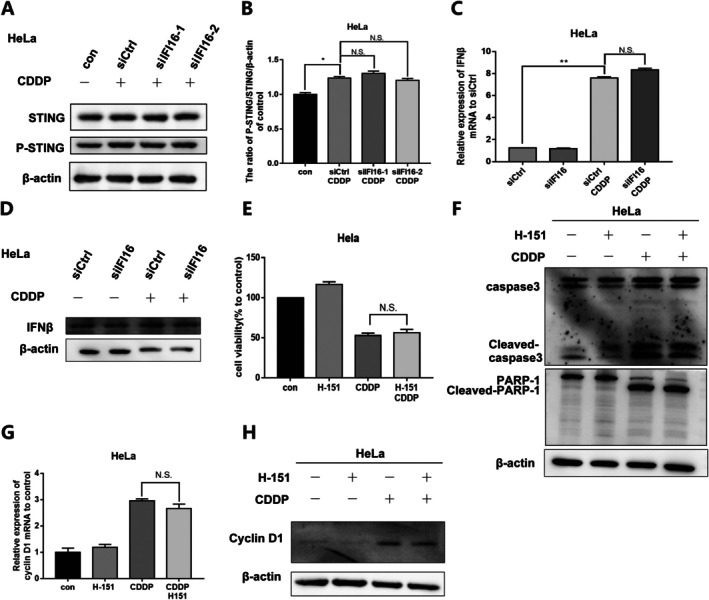
IFI16/NF‐κB resistance to cisplatin induced apoptosis was not related to STING signal. (A) HeLa cells were treated with cisplatin for 12 h after IFI16 knockdown, and the expression changes of STING signalling pathway related proteins P‐STING and STING proteins were detected by Western Blot. (B) Quantitative analysis of protein expression in Figure A. (C) After knocking down IFI16, HeLa cells were treated with cisplatin for 12 h, and the mRNA expression of STING signalling pathway target gene IFN‐β was detected by qPCR. (D) The changes in the expression of IFN‐β protein. (E) After H‐151 pretreatment for 2 h, HeLa cells were treated with cisplatin for 12 h, and the cell activity of HeLa cells was detected by the MTT method. (F) After H‐151 pretreatment for 2 h, HeLa cells were treated with cisplatin for 12 h, and the changes in Cleaved‐caspase 3 protein expression were detected by Western Blot. (G) HeLa cells were treated with cisplatin for 12 h after H‐151 was pretreated for 2 h, and Cyclin D1 mRNA expression was detected by qPCR. (H) The changes in the expression of Cyclin D1 protein were detected by Western Blot. (H‐151:1 μM, CDDP: 4 μg/mL, data described as mean ± SD (*n* = 3), *p < 0.05; **p < 0.01; N.S., *p* > 0.05).

In order to further explore the mechanism of IFI16 antagonising cisplatin action, we investigated whether it activates the NF‐κB signalling pathway through the STING signalling pathway. We used cisplatin to stimulate HeLa cells after IFI16 knockdown and found that DNA damage caused by cisplatin led to activation of the STING signalling pathway. However, compared with the cisplatin group, the p‐STING/STING ratio (Figure 5A,B) and the transcription level and protein expression of IFN‐β (Figure 5C,D), the downstream target gene of the STING signalling pathway, were not significantly changed in the cisplatin group after IFI16 knockdown. These results indicate that DNA damage caused by cisplatin can activate the STING signalling pathway, and cisplatin‐induced activation of the STING signalling pathway has nothing to do with IFI16.

In order to explore the relationship between STING and the antagonism of IFI16/NF‐κB against cisplatin induced apoptosis, we conducted the following experiments using STING inhibitor H‐151. After STING inhibitor H‐151 (1 μM) was pretreated for 2 h, HeLa cells were treated with cisplatin for 12 h. The results showed that there were no significant changes in cell activity and Cleaved‐caspase3 and Cleaved‐PARP‐1 protein expression (Figure 5E,F) in the cisplatin stimulated group after H‐151 treatment compared with the cisplatin alone group. At the same time, no significant changes were observed in the transcription level and protein expression level of CyclinD1 (Figure 5G,H), a proliferation‐related gene of NF‐κB target gene. These results indicated that inhibition of the STING pathway did not affect the sensitivity of HeLa cells to cisplatin, and further demonstrated that STING signalling activated by cisplatin may have nothing to do with IFI16/NF‐κB's resistance to cisplatin induced apoptosis.

These results indicated that the knockdown of IFI16 did not affect the cisplatin‐induced STING pathway activation, and the use of the STING inhibitor H‐151 to inhibit STING signalling did not affect the sensitivity of HeLa cells to cisplatin or the transcription of Cyclin D1, a downstream NF‐κB cell proliferation‐related gene. This suggests that cisplatin‐activated STING signalling may be related to the expression of cisplatin‐induced pro‐inflammatory factors, but is not related to IFI16/NF‐κB resistance to cisplatin‐induced apoptosis.

## 4 Discussion

Cervical cancer is the second most common cancer in women worldwide [1]. Although the HPV vaccine and cervical cancer screening have greatly reduced the incidence of cervical cancer, limited treatment options still result in a poor prognosis. The treatment standards for advanced cervical cancer mainly include radiotherapy and chemotherapy based on cisplatin [26]. Cisplatin treatment is effective in about one‐third of patients with recurrent or metastatic cervical cancer. However, the duration of cisplatin treatment is usually very short, with a median survival of about 1 year [26], indicating an urgent need to improve our understanding of the mechanism of cisplatin treatment for cervical cancer. In this study, IFI16 is induced by p53 into the nucleus and activates the NF‐κB pathway, thus playing a role in the anti‐apoptotic effects of cervical cancer cells in the treatment of cisplatin. Targeted inhibition of IFI16 may be a new way to increase cisplatin sensitivity of cervical cancer cells.

Previous studies have suggested that dsDNA damage can cause the activation of the NF‐κB pathway [13, 14], and p53 plays an important role in the activation of NF‐κB induced by dsDNA damage [15, 16]. Although the more common relationship between NF‐κB and p53 is antagonistic, competing with limited transcriptional co‐activators p300 and CBP [27], multiple experiments have shown that p53 and NF‐κB cooperate and co‐cause changes in gene expression in the context of DNA damage [13]. In addition to regulating the apoptosis of tumour cells, this cooperative relationship may also mediate the enhancement of the immune killing activity of tumour cells. Therefore, the function of the NF‐κB pathway remains controversial. In order to explore the activation mechanism and function of the NF‐κB pathway in the anti‐tumour effect of cisplatin, this study used cisplatin to stimulate human cervical cancer cells HeLa and observed that the protein expression of p53 and p65 in the nucleus was increased with transcriptional activity. To verify the role of p53 in dsDNA damage‐induced NF‐κB activation, we overexpressed p53 using the Pmp53 plasmid in HeLa cells. It was observed that p53 induced p65 into the nucleus and promoted the transcription of its downstream cytokine IL‐6 and Cyclin D1, a gene associated with cell proliferation. These results indicate that dsDNA damage caused by cisplatin can induce the activation of the NF‐κB pathway by inducing p53 and can promote cell proliferation and mediate inflammation. Currently, much attention has been paid to how the activation of NF‐κB by tumour suppressor p53 regulates apoptosis. Previous studies have shown that in tumours that retain wild‐type p53, p53 induces apoptosis by activating NF‐κB via MEK1 [27]. After stimulation of human embryonic kidney cells (HEK) with etoposide, p53 induces pro‐apoptotic protein with death domain (PIDD) to activate NF‐κB to promote or inhibit apoptosis [13, 15]. Thus, differences in upstream regulatory molecules may be the reason for the different roles of NF‐κB pathway.

Studies have shown that IFI16 is closely related to p53 and NF‐κB. To explore the role of IFI16 in the p53/NF‐κB pathway under cisplatin stimulation, we observed increased whole‐cell and nuclear protein expression of IFI16 in the presence of cisplatin‐stimulated p53 upregulation and in the presence of the Pmp53 plasmids. This indicated that p53 induced increased expression of IFI16 into the nucleus. Pmp53 is overexpressed wild‐type p53 plasmids. (For more details about Pmp53 plasmids, please refer to Section 2.3 of the Materials and Methods.) At the same time, we found that the cisplatin sensitivity of cervical cancer cells HeLa was enhanced after knockdown of IFI16, which indicated that IFI16 played a role in resisting cisplatin‐induced apoptosis. This conclusion was verified by using mouse cervical cancer cell U14 to construct a mouse cervical cancer cell subcutaneous implantation tumour model in vivo. To test whether IFI16 is activated to resist cisplatin‐induced apoptosis through the NF‐κB signalling pathway, we found that inhibition of IFI16 reduces cisplatin activation of NF‐κB, which is the main molecule that activates the NF‐κB pathway. It can be concluded that p53 may resist cisplatin‐induced apoptosis in cervical cancer cells by inducing IFI16 to activate NF‐κB (p65).

A number of studies have shown that dsDNA damage activates the innate immune signal STING pathway in human keratinocytes, macrophages and some tumour cells [21]. As a DNA‐binding protein translocating from nucleus to cytoplasm, IFI16 can play the role of intracellular DNA receptor, recognise host dsDNA and other signals from mitochondria or nuclear spillover, and activate STING signalling to regulate NF‐κB [20, 28, 29]. In addition to inducing innate immune response, activation of the STING signalling pathway may also be related to apoptosis. Previous studies have shown that IFI16/STING/NF‐κB signalling recognises exogenous mitochondrial DNA (mtDNA) and increases anti‐apoptotic activity in human endothelial cells (EC) by triggering mitochondrial autophagy [20]. Therefore, the role of the STING signalling pathway in cell proliferation needs further exploration. We observed that cisplatin activated STING signalling in cervical cancer cells is related to cisplatin‐induced proinflammatory factor expression, but may not be related to IFI16/NF‐κB resistance to cisplatin‐induced apoptosis. It is suggested that IFI16 and NF‐κB can be linked through molecules other than STING. Cisplatin‐stimulated IFI16 is mainly located in the nucleus. Therefore, it is speculated that the protein localisation of IFI16 affects the activation mode of NF‐κB.

This study reveals that under DNA damage induced by cisplatin, p53 activates the NF‐κB pathway through nuclear‐localised IFI16, driving cell proliferation (Cyclin D1) and inflammatory response (IL‐6). This study explores the ‘pro‐survival’ function of p53‐NF‐κB that runs parallel to the ‘pro‐apoptotic’ role of the tumour suppressor p53. Notably, the nuclear‐specific localization of IFI16 may enable it to escape the classic STING‐dependent DNA damage response mode and instead reshape the tumour microenvironment through a non‐classical NF‐κB pathway. This study suggests that the p53‐IFI16‐NF‐κB axis under genotoxic stress may be the central hub for tumour cells to achieve dual adaptation of ‘pro‐survival and anti‐apoptosis’, not only recruiting immune cells through inflammatory signals to promote tumour progression, but also maintaining the survival of malignant clones through proliferation signals. This may be the underlying mechanism of cisplatin resistance and tumour recurrence after chemotherapy. This discovery not only expands the theoretical framework of p53 functional polymorphism in DNA damage response, but also indicates that the co‐evolution of inflammation and proliferation in the tumour microenvironment may be a key therapeutic point for regulating chemotherapy sensitivity.

## Author Contributions

**Lili Zhong:** data curation (equal), methodology (equal). **Jiaxin Li:** data curation (equal), project administration (equal), writing – original draft (lead). **Jianfeng Zhong:** formal analysis (equal), methodology (equal), software (equal). **Yifan Zhang:** data curation (equal), formal analysis (equal), supervision (equal). **Hang Qi:** data curation (equal), investigation (equal), supervision (equal). **Huimei Yu:** data curation (equal), methodology (equal), visualization (equal). **Xin Li:** conceptualization (equal), funding acquisition (lead), project administration (lead), writing – review and editing (lead).

## Conflicts of Interest

The authors declare no conflicts of interest.

## Data Availability

The data used to support the findings of this study are available from the corresponding author upon request.
